# Unusual cerebral vascular prion protein amyloid distribution in scrapie-infected transgenic mice expressing anchorless prion protein

**DOI:** 10.1186/2051-5960-1-25

**Published:** 2013-06-19

**Authors:** Alejandra Rangel, Brent Race, Mikael Klingeborn, James Striebel, Bruce Chesebro

**Affiliations:** 1 Laboratory of Persistent Viral Diseases, Rocky Mountain Laboratories, National Institute of Allergy and Infectious Diseases, Hamilton, MT 59840, USA; 2 Department of Ophthalmology, Duke University Medical Center, Durham, NC 27710, USA; 3 Rocky Mountain Laboratories, 903 South Fourth Street, Hamilton, MT 59840, USA

**Keywords:** Brain interstitial fluid, Cerebral amyloid angiopathy, Prion, Glycophosphatidylinositol anchor, Basement membrane

## Abstract

**Background:**

In some prion diseases, misfolded aggregated protease-resistant prion protein (PrPres) is found in brain as amyloid, which can cause cerebral amyloid angiopathy. Small diffusible precursors of PrPres amyloid might flow with brain interstitial fluid (ISF), possibly accounting for the perivascular and intravascular distribution of PrPres amyloid. We previously reported that PrPres amyloid in scrapie-infected transgenic mice appeared to delay clearance of microinjected brain ISF tracer molecules.

**Results:**

Here we studied distribution of PrPres amyloid on capillaries, arteries and veins to test whether vascular specificity of PrPres corresponded to distribution of ISF tracer molecules. To distinguish PrPres-positive arteries from veins and capillaries, scrapie-infected mouse brains were studied by immunodetection of alpha smooth muscle actin. ISF was studied using fluorescein-labeled ovalbumin microinjected into brain as a tracer. In infected preclinical or clinical mice, PrPres was found mostly on capillaries (73-78%). Lower levels were found on arteries (11-14%) and veins (11-13%). Compared to PrPres, ISF tracer was found at higher levels on capillaries (96-97%), and the remaining tracer was found at a skewed ratio of 4 to 1 on arteries and veins respectively.

**Conclusions:**

PrPres association with blood vessels suggested that ISF flow might transport diffusible PrPres precursor molecules to perivascular sites. However, the different vascular specificity of PrPres and ISF tracer indicated that ISF flow did not alone control PrPres dissemination. Possibly blood vessel basement membrane (BM) components, such as glucosaminoglycans, might concentrate small PrPres aggregates and serve as scaffolds for PrP conversion on multiple vessel types.

## Background

TSE diseases or prion diseases are a rare group of slow neurodegenerative brain diseases which affect humans as well as a variety of domestic and wild mammals. In prion diseases normal host-encoded cell surface-anchored prion protein (also known as PrP^C^ or PrPsen due to its sensitivity to protease digestion) undergoes misfolding and aggregation to generate a partially protease-resistant form known as PrPres or PrP^Sc^, which is detectable by immunoblot and is an important biochemical marker for the disease (for reviews see [1,2]). PrPres may also be a causal factor in the associated brain damage observed. Neuropathology of prion diseases in humans and in animals, such as sheep, cattle and rodents, is typically characterized by gray matter vacuolation and gliosis, as well as deposition of PrPres in either a diffuse non-amyloid form or a more dense amyloid form. Diffuse PrPres is the more common form and is seen in human sporadic CJD as well as in most types of animal prion diseases [3-6]. The amyloid form of PrPres is seen in many familial human prion diseases associated with PrP mutations as well as in certain animal prion disease models [7-9].

In mammals PrP is mainly expressed as a glycoprotein anchored to the cell surface by a glycophosphatidylinositol (GPI) linkage. Previously we generated transgenic Tg44 mice, which express only the anchorless form of PrP, and in these mice scrapie-infection results in an unusual type of slow fatal prion brain disease distinguished by widespread deposition of PrPres amyloid in the central nervous system [10] and in extraneural sites such as heart, brown fat, white fat and colon [11,12]. In the CNS of infected Tg44 mice the gray matter vacuolation typical of prion diseases is minimal, and PrPres is primarily deposited as perivascular and intravascular amyloid which is associated with extensive non-vacuolar neuronal damage as well as astrogliosis and microgliosis [10].

The perivascular PrPres amyloid deposition and associated pathology seen in scrapie-infected Tg44 mice is similar to cerebral amyloid angiopathy (CAA) seen in several familial brain amyloid diseases involving mutant proteins or peptides (for review see [13]). In the case of prion diseases, PrP mutations including Y145X, Q160X, Y163X and Y226X [13-16] have been associated with development of CAA with perivascular PrPres amyloid, and all these mutations give rise to truncated PrP molecules which also lack the glycophosphatidylinositol (GPI) anchor. Interestingly, one other human patient expressing PrP Q227X which lacks the GPI anchor had disease with multicentric plaques and no CAA [15]. In humans CAA is most commonly observed with Alzheimer’s disease (AD) where Aβ amyloid is deposited in CNS as both vascular and parenchymal plaques. In CAA associated with Alzheimer’s disease, small arteries and arterioles are the most common sites of Aβ amyloid deposition [13,17,18], but amyloid is also detected in capillaries and in veins at a lower incidence [18-20]. In leptomeningeal vessels of AD patients the ratio of Aβ-positive arteries to veins was 4.5 to 1 [21]. This Aβ amyloid distribution mirrored the pattern of the bulk flow of brain interstitial fluid (ISF) tracers which appear to drain from the brain in perivascular spaces of arteries more than veins [22,23], suggesting that ISF flow might play a role in the vascular distribution of amyloid in AD [24].

In our work with Tg44+/+ mice, we previously observed PrPres amyloid plaques associated with various brain blood vessels which appeared to include arteries, capillaries and veins based on morphological criteria [10]. In the present work using dual staining with anti-PrP monoclonal antibody plus antibody reactive with alpha smooth muscle actin (ASMA) in arteries and arterioles, we studied the vascular specificity of PrPres amyloid distribution in scrapie-infected Tg44+/+ mice. The distribution pattern of PrPres amyloid on blood vessels in this model appeared to be different than that of Aβ amyloid observed in humans with AD, and suggested that PrPres vascular deposition and subsequent CAA might involve a different pathogenic process than that seen in AD.

## Methods

### Experimental mice

Homozygous Tg44+/+ mice expressing a transgene encoding mouse prion protein lacking the GPI anchor (anchorless PrP) were described previously [25]. Mice from 4–6 weeks of age were infected with 1% brain homogenate of RML scrapie stock using intracerebral (IC) or intravenous (IV) routes using volumes of 50 μl and 250 μl respectively as previously described [26-28]. Uninfected age-matched Tg44+/+ mice and non-transgenic C57BL/10SnJ mice were also used in some experiments. All mice were housed at Rocky Mountain Laboratories (RML) in an AAALAC-accredited facility and experimentation followed NIH RML Animal Care and Use Committee (ACUC) approved protocols (protocol # 2010–08).

### Stereotaxic surgery

Intracerebral injections of FITC-OVA were performed on uninfected and scrapie-infected Tg44+/+ mice and uninfected C57BL/10 adult male mice as previously described [27]. Mice weighing approximately 30 g were used to improve accuracy of stereotaxic injection. Scrapie-infected Tg44+/+ mice were injected at 250–280 dpi which was the time of early clinical disease. Uninfected C57BL/10 and Tg44+/+ mice were age-matched to scrapie-infected mice. All injections used 0.5 μl FITC-OVA solution at a rate of 0.25 μl/min. Mice were euthanized 30 min or 7 hours post-injection.

### Primary antibodies

The following primary antibodies were used: rabbit polyclonal antibody anti-alpha-smooth muscle actin (ASMA) (1:500) (Abcam, ab5694). Human D13 anti-PrP recombinant antibody was obtained from tissue culture supernatants made in our laboratory from CHO cells expressing the D13 antibody construct [29], which were kindly provided by Dr. R. Anthony Williamson, The Scripps Research Institute, La Jolla, CA [27]. D13 culture fluid was used at a dilution of 1:100. Primary antibodies were diluted in PBS with 1% normal goat serum and 0.1% Triton X-100. Diluent without antibody was used as a negative control.

### Immunohistochemical staining

Mice were deeply anesthetized with isoflurane and cervically dislocated. Brains were removed, fixed in neutral-buffered formalin (NBF), embedded in paraffin, and processed for immunohistochemical staining for PrPres using D13 monoclonal antibody and detection with DAB chromogen as previously described [26]. Following the DAB step, slides were stained with rabbit anti-ASMA followed by biotinylated goat anti-rabbit IgG (AbCam, Cambridge, MA) (dilution 1:450) and development with avidin-alkaline phosphatase using Fast Red chromogen (Ventana, Tucson, AZ). Hematoxylin was used as a counter stain for nuclei.

### Immunofluorescence staining

Mice were deeply anaesthetized with isoflurane, intracardially perfused with PBS followed by 4% paraformaldehyde in phosphate buffer, pH 7.4, and brains were removed, immersion fixed in 4% paraformaldehyde overnight, cryoprotected, and frozen sections were prepared and stained using the Dako Autostainer Plus (Dako, Carpentaria, CA) as previously described [27]. ASMA staining alone or co-staining with D13 anti-PrP monoclonal antibody followed by anti-ASMA were performed depending on the analysis. Prior to immunofluorescence staining for PrPres, frozen sections on slides were incubated for 15 min at room temperature in 4.0 M guanidine thiocyanate to expose antigenic epitopes of PrPres. Slides were rinsed with diluent and then exposed to D13 for 1 hour at room temperature. After washing with diluent, slides were exposed to Alexa-Fluor 488-tagged secondary goat anti-human Ig antibodies (Molecular Probes, Eugene, OR) for 30 min at a 1:200 dilution. After rinsing with diluent, slides were exposed to rabbit anti-ASMA for 1 hour at room temperature, followed by rinsing with diluent and exposure to Alexa-Fluor 568-tagged secondary goat anti-rabbit Ig for 30 min at a dilution of 1:200. In some cases slides from mice previously injected with FITC-OVA were stained only with anti-ASMA using the protocol above without the guanidine and D13 steps. Nuclei were stained with 0.01% 4′,6-diamidino-2-phenylindole dilactate (DAPI, Invitrogen) for 5 min and rinsed with double-distilled H_2_O, and coverslips were applied to tissue sections with ProLong Gold Antifade reagent (Invitrogen). Slides were examined and photographed either in an epifluorescent Olympus BX51 microscope (Olympus, Center Valley, PA) with Microsuite FIVE software (Olympus) or in a confocal laser-scanning microscope (Zeiss, SLM 510, Carl Zeiss, Germany). For all confocal imaging a Z-stack of 7 to 30 optical sections of 0.38 μm thickness was taken. All images were obtained in sequential scanning laser mode to avoid fluorochrome cross-excitation. Images were managed using Imaris software (Bitplane, So.Windsor, CT).

### Identification and quantitation of blood vessels

**Figure 1:**
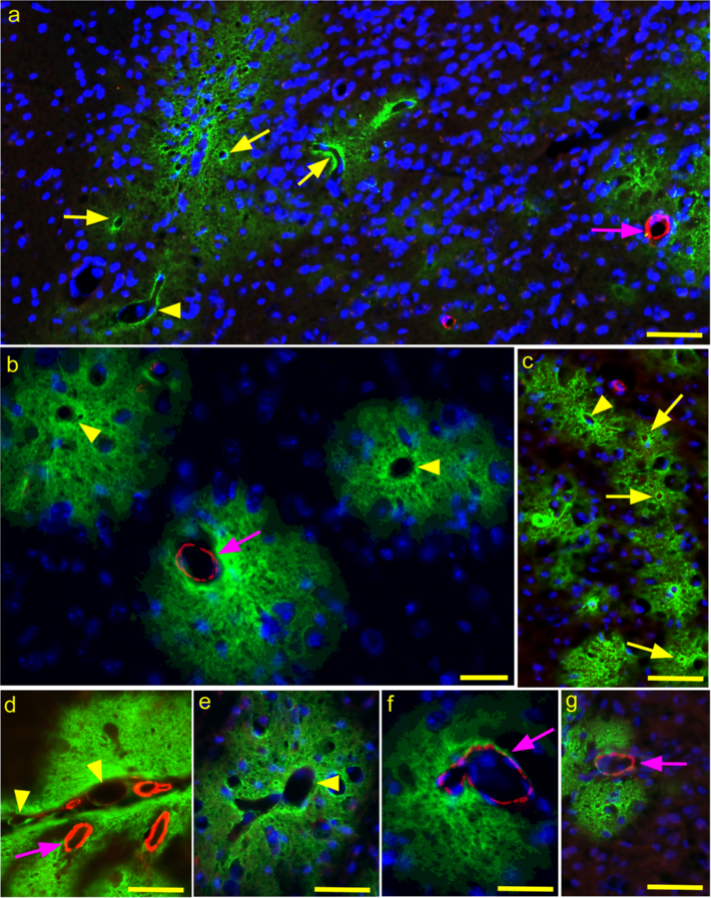
**Immunofluorescence detection of PrPres and ASMA in brain tissue of scrapie-infected Tg44+/+ mice.** Mice were examined at 250–280 dpi which correlates with early clinical signs [10,27]. PrPres was detected with D13 monoclonal antibody (green) and ASMA was detected with rabbit anti-ASMA (red) as described in the Methods section. (**a**) Overview photo showing PrPres plaques (green) and ASMA detection (red). Several capillaries (yellow arrows), one venule (arrowhead) and one arteriole (pink arrow) are shown associated with PrPres plaques. (**b**) Three separate PrPres plaques (green) are seen. One has a central ASMA-positive arteriole (arrow) and the other two have a central vein (arrowheads). (c) Several capillaries (arrows) and one venule (arrowhead) are located in a group of small PrPres plaques. (**d**) PrPres plaques in meninges and adjacent parenchyma associated with 4 ASMA-positive arteries or arterioles (arrow) and two veins (arrowheads).(**e**) PrPres plaque with ASMA-negative vein (arrowhead). (**f** and **g**) PrPres plaques with associated ASMA-positive arterioles (arrows). Bars: 25 μm (**b** and **f**), 50 μm (**a**, **c**, **d**, **e**, and **g**).

Multiple photomicrographs of representative fields were examined to quantitate vessel types as described below. Numbers of mice and total areas examined are given in the table footnotes. Sections were obtained at 110 μm intervals, and photomicrographs including areas of the cerebral cortex, hippocampus, thalamus, hypothalamus and striatum were counted. Blood vessels associated with PrPres or FITC-OVA were identified on the basis of ASMA staining and blood vessel lumen size. The majority of vessels detected were obvious capillaries which were ASMA-negative and had a lumen size ranging from 3-7 μm. ASMA-negative vessels with lumen size 8 μm or larger were considered to be veins or venules. ASMA-positive vessels with thicker walls were considered to be arteries or arterioles. Occasional ASMA-positive vessels had large lumens and thin walls and were scored as veins. An example is shown in Figure 1d.

## Results

### Cerebral vascular distribution of PrPres in scrapie-infected Tg44+/+ mice

To identify the blood vessel types associated with PrPres amyloid plaques in brain, scrapie-infected Tg44+/+ mice were studied during early clinical disease from 250–280 dpi. For immunological studies, brain sections were processed for dual immunofluorescence (IF) using humanized monoclonal antibody D13 for detection of PrPres and rabbit anti-ASMA. Numerous PrPres plaques of varying size were seen in most brain regions. Plaques in Tg44 mice appeared to begin at the blood vessel basement membrane, as seen previously by EM studies [10], and extended into the adjacent parenchyma up to 10–100 microns away from the original blood vessel wall (Figure 1a, b and g).

Arteries, veins and capillaries were identified and distinguished as described in the Methods. We counted photographic fields of brains from 4 mice. Of 152 PrPres-positive vessels initially counted 73% were capillaries, 14% were arteries, and 13% veins (Table 1). The equal incidence of PrPres positive arteries and veins was unexpected as this was not seen with Aβ amyloid in humans with AD or mouse AD models [21,30]. To increase our confidence in these data we studied additional fields in 6 mice counting only the vessels larger than capillaries. Here, out of 140 PrPres-positive large vessels, 43% were ASMA-positive arteries or arterioles and 57% were ASMA-negative veins (Table 2). Thus, PrPres appeared to be associated equally with arteries and veins, but showed a preferential association with capillaries.

**Table 1:** Immunofluorescence detection of PrPres on capillaries, arteries and veins in scrapie-infected Tg44+/+ mice

**Vessels**^**a**^	**N**^**b**^	**%**
Capillaries	111	73
Arteries	21	14
Veins	20	13
Total	152	100
Artery to Vein (A/V) ratio	1.05 to 1	

a - Vessels were defined as described in the Methods section.

b - N = number of PrP-res-positive vessels of each type detected in the total area counted. Total area counted was 7.5 mm^2^ . Data are from 4 individual mice, which were analyzed between 250–280 days post-infection with RML scrapie as described in the Methods.

**Table 2:** Immunofluorescence detection of PrPres on arteries and veins in scrapie-infected Tg44+/+ mice

**Vessels**^**a**^	**N**^**b**^	**%**
Arteries	60	43
Veins	80	57
Total	140	100
A/V ratio	0.75 to 1	

a - Vessels were defined as described in the Methods section.

b - N = number of PrP-res-positive vessels of each type detected. The total area counted was 22.53 mm^2^. Data are from 6 mice which were analyzed between 250–280 days post-infection with RML scrapie as described in the Methods.

**Figure 2:**
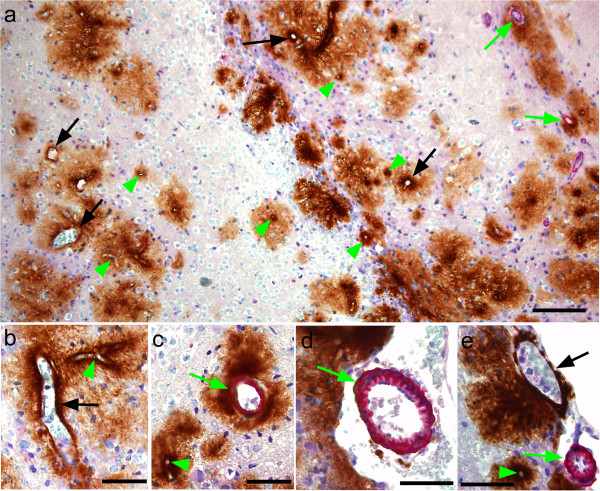
**Immunohistochemical detection of ASMA and PrPres in scrapie-infected Tg44+/+ transgenic mice at 308 dpi. **(**a**) Overview showing perivascular PrPres amyloid plaques (brown) and ASMA (pink) in cerebral cortex. Note association of PrPres plaques with capillaries (green arrowheads), veins (black arrows) and arteries (green arrows). (**b**) Higher magnification of perivascular PrPres on a large ASMA-negative vein (black arrow) and a small capillary (green arrowhead). (**c**) PrPres surrounding an artery (green arrow) and a capillary (green arrowhead). (**d**) ASMA-positive leptomeningeal artery with thin ablumenal ring of PrPres (green arrow). (**e**) Leptomeningeal vein (black arrow) with adjacent PrPres. In lower area a small artery (green arrow) and a capillary (green arrowhead) have associated PrPres. Bars: 100 μm (**a**), 50 μm (**b**-**e**).

The above IF method was very sensitive for detecting ASMA. However, identification of ASMA-negative capillaries and veins was hampered by the lack of a specific positive marker for these types of vessels. Therefore, we also studied scrapie-infected clinical Tg44 mice at 308 dpi using immunohistochemical staining of paraffin sections for PrPres and ASMA where staining with hematoxylin facilitated visualization of nuclei and other tissue structures including vessels walls and allowed easier distinguishing of veins and capillaries. Using IHC staining for both PrPres and ASMA, Figure 2a shows a typical field with many PrPres-positive capillaries, several PrPres-positive veins and venules and two PrPres-positive arterioles. Again many plaques extended quite far into the parenchyma surrounding even small vessels (Figure 2a). At higher magnification ASMA-negative capillaries and veins could be distinguished by size differences, and vessel walls were thin in both cases (Figure 2b). In Figure 2c an ASMA-negative capillary was clearly different from a thicker-walled ASMA-positive small artery. In Figure 2d a larger thick-walled ASMA-positive meningeal artery with a visible elastica layer was located adjacent to PrPres-positive brain parenchyma. In Figure 2e a vein, capillary and arteriole were easily distinguished by ASMA-staining as well as wall thickness and lumen size.

Using this IHC method on paraffin sections, a total of 259 PrPres-positive vessels were counted from three different mice. 78.4% were capillaries, 10.8% were arteries and 10.8% were veins (Table 3). The high incidence of PrPres associated with capillaries and the lower incidence of PrPres on both arteries and veins was very similar to the results seen above using immunofluorescence. The finding of PrPres on all three blood vessel types suggested the possibility that PrPres might spread through the brain by transport via the brain ISF bulk flow as ISF tracers are known to accumulate along the walls of all these vessel types [22,30]. This process might also be enhanced by affinity of PrPres for certain components of blood vessel walls. An alternative explanation for the association of PrPres with blood vessels might be redistribution of PrPres in the brain via hematogenous spread of PrPres seeds.

**Table 3:** Immunohistochemical detection of PrPres on capillaries, arteries and veins in scrapie-infected Tg44+/+ mice

**Vessels**^**a**^	**N**^**b**^	**%**
Capillaries	203	78.4
Arteries	28	10.8
Veins	28	10.8
Total	259	100
A/V ratio	1 to 1	

a - Vessels were defined as described in the Methods section.

b - N = number of PrP-res-positive vessels of each type detected. The total area counted was 12 mm^2^. Data are from 3 individual mice, which were analyzed at 308 days post-infection with RML scrapie as described in the Methods.

### Evidence against hematogenous spread of scrapie infection to brain in Tg44+/+ mice

To study whether hematogenous spread of scrapie PrPres to the CNS occurred in Tg44+/+ mice we compared inoculation of scrapie by intracerebral (i.c.) and intravenous (i.v.) routes. In our previous studies, i.c. scrapie inoculation of Tg44+/+ mice led to appearance of PrPres amyloid in brain and extraneural tissues, including spleen, heart, brown fat, white fat, tongue, skeletal muscle and colon, starting at 150 dpi [11,12], and clinical disease requiring euthanasia occurred from 310-340 dpi [10]. In contrast, i.v. scrapie inoculation of Tg44+/+ mice did not induce any detectable brain PrPres from 150-350 dpi, even though extensive infection of extraneural tissues occurred (Table 4). At later times, from 450-600 dpi, a few mice inoculated by the i.v. route had detectable PrPres amyloid in brain [26]. The route of actual CNS invasion in these mice might have been either via slow neural transport [26] or via blood, if the blood–brain-barrier (BBB) were less efficient at later ages; however, this could not be determined by our experiments. The discrepancy between brain and extraneural infection after i.v. inoculation suggested that the BBB could prevent neuroinvasion of the brain by PrPres from the blood. The protective role of the BBB against hematogenous seeding of brain by PrPres was supported by experiments where a 100% incidence of brain infection was noted after a needle stab wound was made in brain immediately after i.v. scrapie inoculation (Table 4).

**Table 4:** Comparison of induction of PrPres amyloid at 150–350 days post-infection in brain versus extraneural tissues after different inoculation routes

	**PrPres amyloid**^**b**^	
**Route**^**a**^	**Brain**	**Extraneural**
i.c.	10/10	10/10
i.v.	0/6^c^	6/6
i.v. with stab	8/8	8/8

a - Tg44+/+ mice were inoculated with RML scrapie by two routes (i.c, intracerebral; i.v., intravenous) as described in the Methods. For some mice, i.v. scrapie inoculation was followed immediately by a needle stab to the brain to break the blood–brain-barrier. Some of these data were presented previously in a different format [26].

b - Number of mice with PrPres amyloid in brain or extraneural organs (heart, brown fat and colon) detected by immunohistochemistry/total mice inoculated.

c - From 450-600dpi 2 of 4 mice inoculated by the i.v. route had detectable PrPres amyloid in brain. The route of actual CNS invasion in these mice might have been either via slow neural transport or via blood, if the BBB was less efficient at later ages. However, this could not be determined by our experiments.

In summary, hematogenous infection of brain after i.v. scrapie inoculation was a late and infrequent occurrence in Tg44+/+ mice. Thus, hematogenous redistribution of PrPres seeds would not explain the widespread perivascular distribution of PrPres amyloid found in the brains of these mice. In contrast, dispersion of PrPres by ISF flow remained a likely possibility to explain the observed pattern of perivascular PrPres amyloid.

### Analysis of the vascular distribution of a brain ISF tracer in Tg44+/+ mice

We previously studied the interaction between scrapie-induced PrPres and the ISF drainage system in Tg44+/+ mice [27]. In these experiments amyloid PrPres transiently blocked the ISF drainage system as measured by tracers of several different sizes. In contrast, no blockage of ISF drainage was seen in the presence of non-amyloid PrPres in non-transgenic mice. However, in these studies we did not determine the relative distribution of ISF tracer on the different vessels. Therefore, in the present study we determined the vascular distribution of an ISF tracer in Tg44+/+ mice to measure the association of ISF tracer with capillaries, arteries and veins.

**Figure 3:**
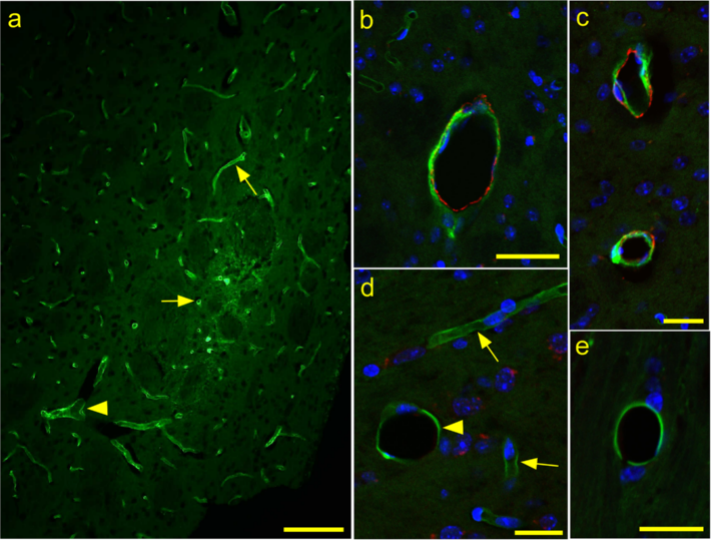
**Immunofluorescence detection of ASMA and FITC-OVA in uninfected Tg44+/+ mice at 30 min after stereotaxic microinjection of tracer.** ASMA is observed as red color and FITC-OVA tracer is green. (**a**) Epifluorescence, (**b-e**) Confocal optical sections of 0.38 μm thickness. (**a**) Most tracer is associated with capillaries (arrows), but some tracer is associated with a larger venule (arrowhead). (**b**) ASMA-positive artery with associated tracer. (**c**) Two ASMA-positive arteries with associated tracer. (**d**) ASMA-negative tracer-positive vein (arrowhead) with 25 μm diameter and two smaller tracer-positive capillaries (arrows) for comparison. (**e**) ASMA-negative tracer-positive vein (17 μm diameter). Bars: 100 μm (**a**), 40 μm (**b**) and 20 μm (**c**-**e**).

Using fluorescein isothiocyanate (FITC)-labeled ovalbumin (OVA) as the ISF tracer, mice were injected with 0.5 μl tracer in the striatum. At 30 minutes and 7 hours post-injection, mice were euthanized and brains were fixed. Cryosections near the injection site were double-stained with antibodies specific for ASMA to identify arteries and arterioles, and sections were examined by immunofluorescence microscopy (Figure 3).

At 30 minutes post-injection of tracer in uninfected Tg44+/+ mice, 96-97% of the tracer-positive vessels were capillaries and the remaining 3-4% were veins and arteries (Table 5). The early high association with capillaries was similar to our previous data where both 5 and 30 minute time-points were studied in uninfected and scrapie-infected non-transgenic C57BL/10 mice and in Tg44+/+ mice [27]. To determine the ratio of tracer association with arteries to veins more large vessels were counted by examining additional microscopic fields in both uninfected and infected Tg44+/+ mice. In these results ovalbumin-positive large vessels in uninfected mice were 82% arteries and 18% veins (A:V ratio = 4.6 to 1), and scrapie-infected mice were similar with 79% arteries and 21% veins (A:V ratio = 3.8 to 1) (Table 6). At 7 h after tracer injection in uninfected mice, tracer was barely detectable in capillaries, but was detectable in arteries and veins at a ratio of 3.1 to 1, which was not significantly different from the ratio seen at 30 min in uninfected Tg44+/+ mice by Fischer’s exact test (P = 0.36). In summary, in Tg44+/+ mice the vascular distribution of the ISF tracer and PrPres differed considerably. Although both FITC-OVA and PrPres showed a high association with capillaries, the percent was significantly higher for FITC-OVA (96-97% versus 73-78%, p < 0.0001) (Table 5). The artery to vein (A:V) ratios were also significantly different for FITC-OVA and PrPres, approximately 4 to 1 for FITC-OVA and 1 to 1 for PrPres (p < 0.0001)(Table 6). Therefore, the ISF flow was not the only factor influencing the distribution of PrPres in infected Tg44+/+ mice.

**Table 5:** Immunofluorescence analysis of FITC-ovalbumin association with capillaries, arteries and veins in uninfected and scrapie-infected Tg44+/+ mice at 30 min after microinjection of tracer

	**Uninfected**		**Scrapie-infected**	
**Vessels**	**N**	**%**	**N**	**%**
Capillaries	1297	96	935	97
Arteries & Veins	50	4	29	3
Total	1347	100	964	100

Data are from 3 mice per group; mice were analyzed between 250–280 days post-infection with RML scrapie as described in the Methods. Area counted: Uninfected = 5.63 mm^2^. Scrapie-infected = 9.01 mm^2^. There was a highly significant difference in numbers of capillaries versus arteries plus veins associated with FITC-OVA (Table 5) versus PrPres (Table 1) in scrapie-infected mice (P < 0.0001) by two-tailed Fischer’s exact test.

**Table 6:** Immunofluorescence analysis of arteries and veins in scrapie-infected and uninfected Tg44+/+ mice for detection of FITC-OVA at 30 min after microinjection of tracer

	**Uninfected**		**Scrapie-infected**	
**Vessels**	**N**	**%**	**N**	**%**
Arteries	78	82	152	79
Veins	17	18	40	21
Total	95	100	192	100
A/V ratio	4.6 to 1		3.8 to 1	

Data are from 3 mice per group; mice were analyzed between 250–280 days post-infection with RML scrapie as described in the Methods. Area counted: Uninfected =10.88 mm^2^, scrapie-infected = 19.52 mm^2^. Results similar to those in Tables 5 and 6 were also observed in uninfected C57BL/10 mice (data not shown). Data for arteries and veins for uninfected versus scrapie-infected Tg44+/+ mice were not significantly different by two-tailed Fischer’s exact test (p = 0.64). In contrast, there was a highly significant difference in numbers of arteries and veins associated with FITC-OVA (Table 6) versus PrPres (Table 2) in scrapie-infected mice (P < 0.0001) by two-tailed Fischer’s exact test.

## Discussion

Scrapie-infected Tg44+/+ mice expressing only the anchorless form of PrP were found previously to deposit PrPres in the CNS as amyloid predominantly in a perivascular and intravascular distribution [10]. This disease required scrapie infection, as it did not occur spontaneously in uninfected Tg44+/+ mice. The lack of the GPI anchor group and the minimal amount of glycan on the anchorless PrP are likely to contribute to the tendency of anchorless PrP to form amyloid, but the reasons for the vascular distribution are not known. In the current studies of the vascular specificity of PrPres amyloid in brain of Tg44 mice, amyloid accumulated mostly on capillaries (73-78%), but also was found on both small arteries (11-14%) and veins (11-13%) (Tables 1 and 3). The association of PrPres with all these blood vessel types could be explained either by hematogenous re-distribution of intracerebrally inoculated scrapie PrPres or, alternatively by dissemination of PrPres via the flow of ISF towards capillaries, small arteries and veins. Our results on studies of mice after intravenous scrapie inoculation did not support the suggestion of hematogenous spread of scrapie infectivity and PrPres (Table 4). Thus, the most likely explanation for the brain perivascular PrPres distribution was dissemination of small diffusible PrPres aggregates by the ISF flow.

**Figure 4:**
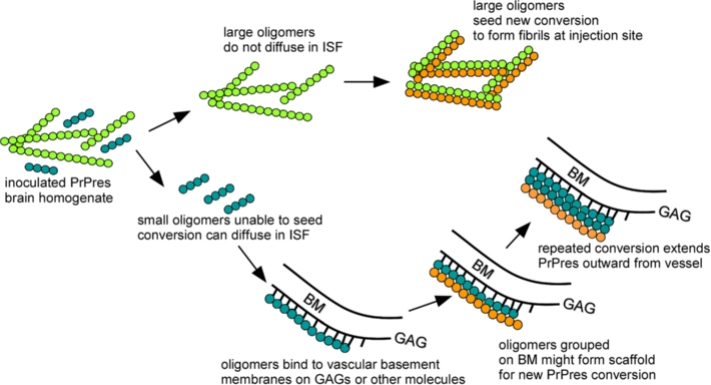
**Cartoon depicting seeding of PrP conversion by fibrils of various sizes in injected scrapie brain homogenate.** Following inoculation larger fibrils (green) might diffuse poorly in brain but would seed PrP conversion (orange) locally near inoculation site. Smaller oligomeric fibrils (blue) may be too small to seed PrP conversion [34], but can travel by diffusion and in brain interstitial fluid (ISF) flow towards blood vessel basement membranes where they might be bound by glucosaminoglycans (GAGs) and other molecules. These concentrated oligomers might form a scaffold capable of seeding new PrPres generation (orange) which in turn might self-scaffold further PrPres conversion (orange) extending both radially and linearly around blood vessel. Smaller oligomers generated at this new site might travel in the ISF flow to new blood vessels and might initiate seeding at these distant sites.

Based on our present finding of PrPres amyloid on capillaries, arteries and veins, amyloid polymerization might be initiated by some BM components, such as glucosaminoglycans (GAGs), found on all of these types of vessels [2,31]. GAGs were previously shown to bind PrPsen [32] and block or potentiate PrP conversion in vitro [33]. Therefore, GAGs in BM might serve as scaffold structures to concentrate, bind and/or align diffusible PrPres aggregates which are too small to act alone as seeds for further PrPres conversion. Presumably after such re-alignment along a scaffold, these aggregates might function effectively as seeds for PrP conversion (Figure 4).

In contrast to the apparent involvement of blood vessel components in PrPres amyloid formation, it was surprising that amyloid plaques in this model often extended large distances (10 to 100 μm) away from the ablumenal blood vessel walls. Possibly, after establishment at the BM, PrPres amyloid might act without GAGs to self-scaffold further conversion of anchorless PrP at locations quite distant from the blood vessel BM. This conversion might also be increased by the abundance of diffusible anchorless PrP which is secreted into the extracellular space by many CNS cell types. In addition, anchorless glycan-negative free-floating PrP in extracellular spaces might have a greater tendency to form amyloid compared to normal membrane-anchored glycan-rich PrP [2].

Anchorless PrP, similar to that found in Tg44 mice, has been detected in association with several different human PrP mutations in patients with familial prion disease. In these cases, mutation of one PrP allele results in aberrant C-terminal PrP truncation (Y145X, Q160X, Y163X, Y226X, and Q227X) with production of shortened PrP lacking the GPI anchor moiety [13-16]. In these patients PrP amyloid with CAA is often a predominant neuropathological finding, and in the figures describing the Y145X and Y226X patients, the vascular specificity of the CAA appears to involve capillaries, arteries and veins. This was similar to the Tg44 model, except that in Tg44 mice capillary amyloid was more prominent (Tables 1 and 3). With all the above-mentioned human PrP mutations, the plaques appeared to be located in blood vessel walls. In addition, in the publications describing both the Y145X and Y226X mutations, the plaques also extended up to 15-35 μm into the parenchyma from the ablumenal vessel wall. This was similar to infected Tg44 mice where the PrPres amyloid plaques extended 10-100 μm into the surrounding brain tissue (Figure 1a-g and 2a-e). Thus, based on vascular specificity, plaque size and plaque location, neuropathology in humans with Y145X and Y226X was similar to that seen in scrapie-infected Tg44 mice.

CAA has also been extensively observed in association with Aβ amyloid deposits in Alzheimer’s disease patients, and CAA may be an important aspect of the neuropathogenesis of AD [17,20,24,35-38]. Interestingly, CAA in these patients differs from infected Tg44 mice and from that seen in the Y145X and Y226X human PrP truncation mutations described above. First, the amyloid in Aβ CAA is mostly located within or very close to the vessel walls [13,20], whereas PrPres amyloid appears to extend quite far away from the vessel wall [10,14,15]. Second, in Aβ CAA the vascular specificity of the amyloid deposition shows a preferential association with small arteries compared to veins which is not seen in PrPres CAA. For example, in the leptomeninges of AD patients, in one study small arteries and arterioles appeared to be the site of 80% of the amyloid, whereas only 20% was associated with veins [21]. However, in a different paper, the amyloid distribution in leptomeningeal vessels was arteries 56% and veins 45% [19]. Therefore, there may be variation in this ratio among different patients. In the cerebral cortex small arteries have been described as the main site of amyloid deposition, with lesser amounts of amyloid associated with veins, venules or capillaries [20]. However, in a subset of AD patients with CAA, designated Type 1-CAA, cortical Aβ amyloid was associated with capillaries, arteries and veins [19], and a role for capillary CAA in specific clinical dysfunction has been suggested [39,40]. Thus, based on the vascular amyloid distribution, scrapie-infected Tg44 mice appear to be somewhat similar to the group of CAA-type 1 Aβ patients.

The reasons for the vascular specificity of CAA involving various different amyloid proteins are not known. Perhaps different proteins have differing requirements for BM-derived co-factors for amyloid formation. If Aβ amyloid were unable to self-scaffold efficiently in vivo so that a blood vessel-derived co-factor was always required, Aβ amyloid deposition might be more restricted to the blood vessel wall and less likely to extend out into the parenchyma. Similarly, if vascular smooth muscle cells, rather than BM, were the source of the essential co-factor(s), this might restrict deposition of Aβ amyloid to the walls of small arteries and arterioles which have abundant smooth muscle cells, as opposed to veins which have few smooth muscle cells. Perhaps capillary-derived co-factors are involved in Aβ CAA type 1 where capillary CAA is prominent [19].

The differences in PrPres and Aβ plaque size and vascular distribution might also be influenced by numerous other factors, including the rates of association, refolding and dissociation of the amyloid complexes in different sites, and the ability of glial cells and macrophages to breakdown the amyloid. The large difference in size between Aβ (40–42 amino acids (aa)) versus anchorless PrP (210aa in Tg44, 204aa in Y226X, and 120aa in Y145X), might also act by influencing some of these mechanisms. Single amino acid sequence differences can also influence the vascular specificity of amyloid deposition, as seen by differences between the effects of Y226X and Q227X PrP mutations, where the former is associated with CAA and the latter has parenchymal multicentric plaques without CAA [15]. However, the mechanisms of most of these effects remain unknown.

Many previous studies have followed clearance of ISF tracers labeled with radioactive or fluorescent tags (for review see [22,30,41,42]). In our previous work, tracer was detectable on blood vessels at 30 min, much reduced at 7 hours and almost completely gone by 24 hours [27]. The present study provides quantitative data on specificity of the ISF tracer FITC-OVA for capillaries, arteries and veins. At 30 min post-injection of Tg44 mice we found the majority of tracer was associated with capillaries (96%), whereas arteries and veins were much lower (3.2% and 0.8% respectively) (Tables 5 and 6). It was not surprising that capillaries were the primary site of tracer location at early times post-injection because capillaries are the most numerous blood vessels in the brain. ISF tracer association with capillaries and arteries has been described previously by several groups [22]. In contrast, tracer association with veins was inconsistent in different systems. Some reported no association of ovalbumin or 3 kD dextran with veins [22]; however, another paper by this same group using 10 kD dextran reported a significant association with veins [30]. In the present studies, we found an artery to vein (A/V) ratio of approximately 4 to 1 for non-capillary-associated tracer at 30 min post-injection (Table 6), and at 7 hours post-injection this ratio was 3.1 to 1, which was not significantly different from the 30 min time-point. Thus, approximately, 20-30% of the tracer associated with larger vessels was associated with veins, and this tracer appeared to be cleared from the CNS by a mechanism involving veins.

The explanation for the skewed A/V ratio in our tracer clearance results remains unclear at the present time. ISF flow is thought to be driven mainly by pressure due to production of new ISF by brain capillary endothelial cells [41]. Although some of the ISF appears to drain via diffusion into the CSF [42], it is believed that much of the perivascular ISF is collected by lymphatic vessels surrounding blood vessels as they emerge from the skull [22,23]. This explanation for outflow and collection of brain ISF outflow could be applicable equally to arteries and veins. The 4 to 1 A/V ratio seen in our experiments may relate to the amount of BM space available around endothelial and smooth muscle cells in vessels where the ISF flows. The larger amount of BM associated with smooth muscle cells in walls of arteries compared to veins might provide an increased space for ISF flow out of the brain on arteries versus veins.

Another explanation for the prominent detection of ISF tracers on arteries versus veins suggests that the contrary (or reflection) wave traveling in the reverse direction from the pulse wave created by the heart beat might drive the ISF along the outside of arteries in the opposite direction to the blood flow [23,43]. The absence of valves along the ISF path outside blood vessels may be a problem for this option. However, of equal importance, this proposal would not explain the clearance of a substantial amount of ISF tracer associated with veins.

The relative selectivity of ISF tracer for arteries versus veins of 4 to 1 observed in our studies is in close agreement with the 4.6 to 1 ratio for Aβ association with arteries versus veins in leptomeninges of humans with AD [21]. Therefore, our results support the previous conclusion by Weller and co-workers that the vascular specificity of ISF flow may be an important factor in the accumulation and distribution of vascular Aβ amyloid in AD [24,38]. In contrast, our present data on vascular distribution of PrPres amyloid suggested that ISF flow might be one of several factors involved in perivascular PrPres distribution. Other factors, such as vascular BM-derived co-factors for amyloid scaffolding, might also be important in vascular specificity of PrP amyloid both in the Tg44 mouse model and in humans with familial prion disease related to expression of mutated truncated PrP.

## Conclusions

Vascular distribution of PrPres amyloid in scrapie-infected Tg44+/+ mice expressing anchorless PrP differed from the vascular distribution of ISF tracer molecules followed in these same mice. Thus, although ISF flow appeared to mediate the transport of small molecules such as PrP oligomers towards blood vessels, the ISF flow alone did not completely determine the vascular deposition of PrPres amyloid. We hypothesize that other factors including basement membrane components such as glucosaminoglycans and laminin, might act as scaffolds to increase amyloid formation by PrP or other proteins starting at the vascular basement membrane (Figure 4). Such scaffolding molecules might differ on different blood vessel types, and the requirements for such factors might differ among different proteins forming amyloid. These factors might account for the varying vascular specificities observed in different CAA diseases in mice and in humans.

## Competing interests

The authors declare that they have no competing interest regarding data presented in this manuscript.

## Authors’ contributions

Experiments were done by AR, BR, and MK. Data were analyzed by AR, JS, and BC. Paper was written by AR, BR, JS and BC. All authors read and approved the final manuscript.
